# The Role of Servant Leadership in Fostering Psychological Safety Among Nurses in Jordanian Hospitals

**DOI:** 10.1155/jonm/1028249

**Published:** 2025-06-17

**Authors:** Luma Asfour, Ali Moh'd Saleh, Yousef Abu-Wardeh

**Affiliations:** ^1^School of Nursing, The University of Jordan, Amman 11942, Jordan; ^2^Clinical Audit Division, Institutional Development and Quality Control, Ministry of Health, Amman, Jordan; ^3^Department of Clinical Nursing, Faculty of Nursing, Zarqa University, Zarqa 132222, Jordan

**Keywords:** critical care nurses, Jordan, psychological safety, servant leadership

## Abstract

**Background:** Servant leadership (SL), characterized by moral behavior and a focus on serving and empowering team members, is particularly relevant in a critical care setting, where a highly pressured environment requires a consistent and empowered team. Psychological safety (PS), a high perception level, and speaking without fear of penalty are highly recommended in critical care areas, including ICUs, CCUs, ERs, burn units, and any other departments that require intensive care for patients to maximize professionalism and improve patient outcomes.

**Aim:** This study aimed to investigate the relationship between SL and PS as perceived by nurses in critical care areas of Jordanian hospitals.

**Method:** A descriptive, cross-sectional, and correlational design was used. A convenience sample of 207 registered nurses working in inpatient departments and the Hemodialysis Unit across five Jordanian hospitals participated in the study. Data were collected using the SL survey and the PS survey to measure the nurses' perceptions of SL and PS.

**Result:** Critical care nurses reported a moderate perception of SL (*M* = 66.7 out of 108) and PS (*M* = 90.4 out of 133). A strong, significant, and positive correlation was found between leadership and PS (*r* = 0.74). Regression results indicated that SL was a significant predictor of PS (*β* = 0.82, *p* < 0.001).

**Conclusion:** SL is a key factor in healthcare institutions that facilitate PS among critical care nurses.

## 1. Introduction

Healthcare institutions focus on improving health, and nurses form the cornerstone of these institutions. Efficacious nurse leaders play a crucial role in promoting the health and well-being of nurses, supporting their team members, and creating an environment where they can openly express their feelings. Treating team members fairly and fostering their skills and knowledge are essential to enhancing patient-centered care [1]. Healthcare institutions should enhance their healthcare providers (HCPs) to achieve optimal outcomes [2]. Leaders are critical to institutional success; their characteristics necessitate unique training, as they are responsible for shaping work habits, fostering positive team performance, and distinguishing between thriving teams and those with internal conflicts and poor productivity [3].

### 1.1. Servant Leadership (SL)

SL is one of the leadership styles that prioritizes the needs of followers and inspires others' enjoyment [4, 5]. SL, as defined by the father of SL Greenleaf in 1970, prioritizes serving first. It emphasizes empowering followers rather than controlling them, thereby fostering organizational growth and creativity [6, 7]. Greenleaf also claimed that the SLs are supportive and problem solvers. Moreover, it stimulates others to develop their personalities [8]. Dierendonck et al. [9] identified five dimensions of SL: empowerment, standing back, humility, authenticity, and stewardship. SLs prioritize followers' needs and promote their growth and development. Servant leaders focus on the psychological aspects of their followers, empowering them and serving others [10]. Nurse leaders in critical care units play a vital role in fostering a nonpunitive culture that promotes open communication. They empower nurses through knowledge and practice enhancement, inspiring them to innovate and increase patient and nurse satisfaction while ensuring the delivery of high-quality intensive care [3, 11].

A servant leader is service-oriented and follower-oriented, in addition to empowering others and prioritizing their needs [12]. On the contrary, ineffective leaders are self-oriented, humiliating and egotistic persons who have destructive behaviors that negatively affect nurses, forcing them to work under pressure, leading them to have emotional distress and performing in an unhealthy environment that decreases nurses' creativity and improvement, undesirably affecting the organizational goals and achievement [13]. SL supports nurse leaders in empowering nurses and fostering a positive work environment [14]. Additionally, it creates learning opportunities by enhancing communication and reporting of adverse events [15].

Greenleaf mentioned that the SLs' characters include inspiring, listening before judging, advising, respecting, stimulating, positively influencing, feeling, empathizing, supporting juniors and treating them with ethics and values [16]. They also build professional social relationships and teamwork with followers, are aware of their surroundings, and understand themselves. This creates a healthy environment, enables them to predict upcoming events, and empowers them to make decisions and solve problems. They are responsible for their decisions and are always behind the scenes, focusing on achieving institutional goals. Moreover, they understand others' strengths and weaknesses [8, 17–20].

Research on SL in healthcare settings has revealed its significant positive impact on nurses' perceptions and job performance across various countries such as Istanbul, China, Pakistan, and India [21–27]; moreover, one study in Jordan consistently shows moderate-to-high perceptions of SL among nursing staff, with mean scores ranging from 3.29 to 5.92 out of various scales [14]. Overall, the findings demonstrate the importance of SL in enhancing nurses' perceptions and performance within healthcare organizations.

SL enhances positive employee outcomes through employee voice and feedback. Moreover, PS has a mediating role in increasing these outcomes [2]. Leaders play a critical role as servant leaders in creating conditions that impact psychological safety (PS) among junior healthcare professionals, as their leadership styles significantly influence the psychological well-being of these professionals [15]. Servant leaders develop followers, promote moral and ethical principles, and encourage followers to become effective leaders. Moreover, they focus on the psychological aspects of others [10].

The Jordanian healthcare system has experienced significant advancement recently, improving its services, technological capabilities and educational resources. However, a thorough analysis of the healthcare system is still lacking. The healthcare environment in Jordan faces challenges. These challenges include the patient–nurse ratio, nurse burnout, and bedside nurses' dissatisfaction [28]. Moreover, the lack of a clear leadership style in Jordan's healthcare system negatively impacts employee satisfaction and patient care. Leaders' styles, such as the high-power leadership style or leaders who adopt a humble ownership approach, also negatively impact employee satisfaction and patient care. In contrast, embracing SL can significantly enhance commitment and create a positive work environment. This style empowers healthcare professionals, improves patient outcomes, and fosters open communication, allowing staff, particularly nurses, to voice concerns and address workplace issues effectively, ultimately leading to better quality of care [14]. This suggests that the effectiveness of SL in promoting PS may be particularly salient in contexts like Jordan, where organizational hierarchies are strong and leadership style plays a critical role in shaping team dynamics. Another study conducted in Jordan examined the relationship between SL and nurse turnover, revealing a negative impact and a direct link to nurse empowerment in the workplace [29].

### 1.2. PS

Edmonson and Lei [30] defined PS as “to feel safe at work to grow, learn, contribute, and perform effectively in a rapidly changing world.” PS is a dynamic phenomenon that examines employees' perceptions of promoting communication, sharing ideas without fear and speaking up about problems and mistakes during work freely [31], developing teamwork, team voice, thinking critically, improving patient safety, and giving alternative ideas [32, 33]. Moreover, it enables juniors to request help and correct their mistakes in critical care situations without feeling unsafe while also fostering confidence [15, 34].

Patient safety, the learning process, teamwork, motivation, and performance, as well as decision-making and problem-solving, are positively or negatively impacted by PS in HCPs [33]. PS is a vital part of nurses' role as a follower resource in healthcare institutions to enhance their self-esteem [21]. PS encourages communication and discussions between team leaders and HCPs, speaks up about their errors and malpractice without fear of retribution, and fosters a culture of accountability to improve the quality of care [32].

The healthcare institutional hierarchy, which categorizes positions into senior and junior categories, is a barrier to PS; the seniors freely speak up about their concerns and errors, while juniors are restricted from speaking about their needs and concerns; these differences lead to reduced communication between them, minimize learning and sharing experience, consequently, inhibit juniors creativity feels insecure [32]. Moreover, juniors have concerns about working in stressful environments and being hesitant to speak up about work problems, especially in front of untrusted peers, which also affects their career development and empowerment [35]. Healthcare professionals may experience apprehension when expressing their opinions and making decisions within their institutions due to fear of negative consequences or a lack of a supportive environment; managing these apprehensions empowers HCPs to contribute their insights and ideas toward the ultimate goal of providing high-quality care for patients [16]. Furthermore, the culture of healthcare institutions punishing HCPs who report adverse events tends to discourage HCPs from reporting incidents due to the consequences of punishment; this can lead to a lack of transparency and accountability, ultimately affecting the quality of patient care [36].

A review of several studies conducted in Spain, China, and Ghana reveals a generally moderate perception of PS among healthcare professionals and nurses, with varying mean scores reported across different contexts. Research indicates that PS plays a crucial role in enhancing team performance, fostering open communication, and encouraging error reporting, ultimately improving patient safety and care quality. Factors such as leadership, teamwork, and work environment have a significant influence on PS levels, with evidence indicating that higher PS is associated with increased job satisfaction and participation in quality improvement efforts. Qualitative insights reveal that supportive leadership is essential in promoting PS, enabling healthcare professionals to voice concerns without fear and thereby fostering a culture of transparency and safety [21, 32, 37–41].

A study conducted in critical care units across 15 hospitals in Korea, involving 200 emergency nurses, explored the relationship between PS and patient safety competency. The findings indicated that nurses had a moderate perception of PS, which positively correlated with their safety competency. A greater perception of PS was associated with improved error reporting and adherence to safety checklists. However, concerns were raised regarding patient-related activities, and the nurses had a limited understanding of the patient safety culture. Factors such as high workload and situation monitoring had a negative impact on PS, whereas teamwork and communication were associated with better safety outcomes [38].

### 1.3. The Relationship Between SL and PS

Several studies have examined the relationship between SL and PS in healthcare settings, with a particular focus on nurses. Overall, these studies emphasize the significance of SL in enhancing nurses' PS and well-being within healthcare organizations. Moreover, PS is vital to mediate the impact of SL on nurses' burnout, reinforcing the benefits of a supportive work environment [3, 12, 21, 42].

A study conducted in Pakistani hospitals, focusing on nurses dealing with COVID-19 patients, revealed that both SL and PS were inversely related to nurses' burnout during the COVID-19 pandemic [43].

Many researchers have demonstrated that SL promotes PS among nurses in all nursing departments, particularly in critical care units, which in turn fosters a healthy work environment in healthcare institutions. For instance, one study highlighted the positive impact of SL in cultivating PS within the Chinese nursing context. Moreover, the study revealed that PS mediates the relationship between SL and nurses' turnover and burnout, indicating that a supportive leadership approach benefits not only employees themselves but also maintains the overall success of the organization [21]. Another study confirmed the crucial role SL plays in improving employee PS [41].

Many studies affirm a positive relationship between SL and PS [3, 12, 42, 43]. However, limited research has examined these dynamic variables among Jordanian nurses. Moreover, few studies have critically analyzed the relationship between SL and PS, specifically under specific conditions such as a high-stress healthcare work environment. This study examines the significance of SL among nursing leaders in Jordanian hospitals, aiming to investigate its relationship with nurses' PS in critical care units. Since nurses make up a unique cultural and professional context, are the largest segment of HCPs in Jordan, and frequently encounter high-pressure situations, this study is particularly relevant.

## 2. Methods

### 2.1. Design

A descriptive, cross-sectional, correlational design was used in the methodology surveys.

### 2.2. Study Setting and Participants

The data for the current study were collected from registered nurses in various critical care units, including emergency departments (ER), intensive care units (ICUs), cardiac care units (CCUs), burn units, inpatient departments, and the hemodialysis unit, using convenience sampling from five hospitals. The researchers selected this method of sampling due to its suitability for collecting preliminary data within the limitations of the clinical environment. This method facilitated timely data collection. Moreover, the exploratory nature of the study allowed for the collection of diverse nurses' demographic data. Finally, factors such as time, cost, and accessibility further warranted the use of the convenience sampling method.

The hospitals were selected in the central region of Jordan, with a total bed capacity exceeding 200 beds, and serve at least 10 medical specialties in each hospital. The five hospitals varied from governmental to private and university-affiliated hospitals. The selected nurses meet the following criteria: they have at least 1 year of experience in critical care units and provide direct patient care in a full-time capacity. Nurses who had administrative responsibilities were excluded from this study. Nurses with an administrative role were excluded to maintain a clear focus on how SL influences the PS of frontline bedside nurses directly involved in patient care.

### 2.3. Sample Size Estimation

The sample proportions were computed using G^∗^ power 3.1 software, based on the following criteria: a significance level of *α* = 0.05, an estimated power (1 − *β*) of 0.95, and a medium effect size of *r* = 0.15. The estimated sample size is 184 participants. The researchers anticipated a 10% dropout rate of participants. Ultimately, the total sample consisted of 207 participants.

### 2.4. Measures

The measurement tools were a self-reporting survey in English consisting of three sections. The existing literature guided the analysis of socio-demographic variables. They consisted of gender, age, marital status, level of education, work area, years of experience, and hospital type [41, 44].

Secondly, a short version of the SL survey measurement tool was used to assess the nurses' perception of SL behaviors; this survey was adopted and approved [9]. The tools consisted of 18 items and used a six-point Likert scale, where selecting one indicated “strongly disagree” and six indicated “strongly agree.” It is divided into five subscales: empowerment (6 items), humility (3 items), standing back (3 items), authenticity (3 items), and stewardship (3 items). The total score for each subscale was as follows: for empowerment, 6–36, and all other subscales, 3–18. The total average scoring ranged between 18 and 108, and a higher average score reflected a stronger SL.

Factorial validity was employed to establish the construct validity of the short version of the SL survey. In addition, reliability was measured by taking the correlation between items. Cronbach's alpha for each subscale was 0.91 for empowerment, 0.91 for humility, 0.86 for standing back, 0.91 for stewardship, and 0.89 for authenticity. The internal consistency for the total scale was 0.95. In conclusion, the internal consistency for each subscale and the full scale indicates that this instrument was reliable and valid [9]. The data were classified into equal-length categories using class intervals ranging from low (< 48), moderate (48–78), and high levels (> 78) depending on the formula: class interval = (range of data)/(number of class intervals) as reported by [45].

The third measurement tool was the PS survey. The survey consists of 19 items of the PS survey and is divided into three sections. The first section was related to the relationship with team leaders and contained nine items, ranging between 9 and 36, while the second was related to peers and contained seven items, ranging between 7 and 49; and the third section was related to the “team as a whole, ranging between 3 and 21.” The seven-point Likert scale was used, where selecting one indicated “strongly disagree” and seven indicated “strongly agree.” The total survey score average ranged from 19 to 133. A higher score indicated a higher PS level; this measurement was adopted and approved by O'Donovan [33].

Previous studies have shown that surveys are more comfortable and indicate high PS in teams; however, interviews revealed issues such as junior workers remaining hesitant to take interpersonal risks and a fear of punishment [15]. Tool reliability was measured section by section; the total score Cronbach's alpha for the team leaders' section was 0.953; the peers' section was 0.955; and the whole team section was 0.791. Cronbach's alpha of the total scales was 0.930. In conclusion, the internal consistency for each subscale and the full scale indicates that this instrument was reliable and valid [33]. The data were classified into equal-length categories using class intervals ranging from low (< 57), moderate (57–95), and high levels (> 95) depending on the formula: class interval = (range of data)/(number of class intervals) as reported by [45].

In the current study, a pilot test was conducted on 20 participants, demonstrating the reliability of these tools. The SL total score Cronbach's alpha was 0.961, and each subscale had the following alphas: humility, 0.876; authenticity, 0.81; stewardship, 0.921; and standing back, 0.733. PS Cronbach's alpha was 0.943, and the subscales were 0.952, 0.937, and 0.915 for “in relation to the leader,” “in relation to peers,” and “in relation to the team as a whole,” respectively.

### 2.5. Data Collection

The researchers visited each hospital and distributed surveys to eligible nurses using a Google Forms link. The surveys were designed to take approximately 10–15 min to complete. Using “Google Forms” enables researchers to customize their surveys by asking numerous questions, controlling answer choices, and regulating criteria based on research interest. Moreover, the widespread use of smartphones makes it easier for participants to complete the surveys at their convenience and simplifies the process of entering data directly into an application that is used for data analysis [46].

However, ensuring data accuracy and integrity, as well as preventing data publication, are significant concerns when using electronic surveys. To address these issues, researchers eliminate duplicate email addresses for privacy. After removing duplicate email addresses, the researchers remove all email addresses for privacy. Subsequently, “Google Forms” allows the researchers to limit the number of participants' responses per email to prevent duplication. Then, the method of answering the question does not permit the participants to miss any questions. Finally, a researcher used a random letter called (captcha) to appear before filling out the survey to be sure it was a human and not a bot [47].

### 2.6. Ethical Considerations

The Institutional Review Board at the University of Jordan granted ethical approval for the conduct of this study, and approval for data collection was also obtained from the hospitals. Prior to signing the implied consent form, all participants were informed of the study's objectives and procedures. They were also informed that they had the right to withdraw from the study at any time without facing any negative consequences. Data were collected using Google Forms, and all information was kept confidential. The survey responses were assigned numerical codes, and only the primary researcher was authorized to access these data. All study data and participants' information were kept in a secure and confidential area; only the researchers can access these data for this study. The researchers removed all email addresses for privacy.

### 2.7. Data Analysis

Data were initially entered into Excel and then exported to SPSS version 26 for accuracy checks and analysis. Descriptive analysis was conducted to calculate means, standard deviations, frequencies, and percentages for the study variables, describing the demographic characteristics of the participants and their perceptions of SL and PS. The Pearson *r* correlation coefficient was used to explain the relationship between variables. One-way ANOVA and *t*-tests were employed to identify differences between variables. Standardized regression analysis was conducted to assess the extent to which various demographic factors and SL behaviors predict variations in PS levels.

## 3. Result

### 3.1. Sociodemographic Characteristics of the Study Participants

The study involved 207 registered nurses with a diverse range of demographic characteristics. Most participants were aged 31–35 years (33.3%), followed by those aged 36–40 (22.2%). The smallest group, over 41 years old, accounted for 12.6%. The majority of nurses were women (63.8%), while men comprised 36.2%. Regarding educational attainment, 77.3% held a bachelor's degree, 21.3% held a master's degree, and only 1.4% held a PhD. In terms of professional experience, all nurses had over 1 year of experience. Specifically, 29.5% of nurses had 6–10 years of experience, while 24.2% had either 1–5 years or 11–15 years of experience, and 22.2% had more than 15 years of experience.

Nurses primarily worked in various hospital units, with the highest percentages in the ICU (22.7%), the ER (14.5%), and medical wards (10.1%). Other areas, such as surgical wards, NICUs, gynecologic and obstetric units, and operation rooms, each accounted for smaller proportions ranging from 6.3% to 9.2%. Less frequently used working areas included CCU, hemodialysis, pediatrics, oncology, endoscopy, and catheterization labs, each accounting for 4.8% or less. Lastly, the distribution of participants was evenly divided (33.3%) among three types of hospitals: governmental, university-affiliated, and private, providing a reassuring balance across different hospital types.

### 3.2. SL Survey Descriptive Statistics

The descriptive statistics for the SL Survey encompassed 207 participants (Table 1). The overall SL score had a mean of 66.7 (SD = 18.4), with scores ranging from 18 to 108. Among the subscales, empowerment showed the highest mean score of 22.8 (SD = 6.9), followed by Stewardship (*M* = 11.2, SD = 3.6), Standing Back (*M* = 11, SD = 3.2), Humility (*M* = 10.8, SD = 3.5), and Authenticity (*M* = 10.8, SD = 3.7). Items within these subscales revealed moderate agreement, with most individual items averaging around 3.6–3.9 on a 6-point scale. Median scores (MD = 4) and interquartile ranges (P25 = 3, P75 = 5) indicate consistent responses, with managers being perceived as moderately effective in providing support, fostering learning, embracing feedback, prioritizing collective well-being, and demonstrating authenticity.

### 3.3. PS Survey Descriptive Statistics

The descriptive statistics for the PS survey encompassed 207 participants (Table 1). The total PS score had a mean of 90.4 (SD = 21.8), with scores ranging from 19 to 133. Participants rated PS in three key areas: in relation to their team leader (*M* = 42.7, SD = 11.5), peers and team members (*M* = 33, SD = 8.6), and the team as a whole (*M* = 14.7, SD = 3.9). Individual items across all areas scored moderately high, with means ranging from 4.5 to 5.7 on a 7-point scale.

PS with team leaders was reflected in strong agreement on asking questions (*M* = 5.7, SD = 1.6) and sharing opinions (*M* = 4.9, SD = 1.6). Similar trends were observed among peers, with participants feeling safe to express their opinions (*M* = 4.9, SD = 1.3) and acknowledge mistakes (*M* = 4.5, SD = 1.7). At the team level, ease in asking for help (*M* = 5, SD = 1.5) and efforts to share information (*M* = 4.9, SD = 1.5) were highlighted. Median scores (MD = 4-5) and interquartile ranges (P25 = 3–6, P75 = 5-6) suggest a generally positive perception of PS within nursing teams.

### 3.4. The Correlation Between SL and PS

The analysis (Table 2) reveals significant positive correlations among SL, PS, and their respective subscales. Total SL exhibits a strong positive relationship with total PS (*r* = 0.74, *p* < 0.001) and with its subscales: PS in relation to the team leader (*r* = 0.77, *p* < 0.001), peers (*r* = 0.57, *p* < 0.001), and the team as a whole (*r* = 0.61, *p* < 0.001). Among the dimensions of SL, empowerment shows the strongest correlations with total PS (*r* = 0.68, *p* < 0.001) and PS in relation to the team leader (*r* = 0.71, *p* < 0.001). Humility and standing back also demonstrate notable, albeit slightly weaker, correlations with PS, ranging from 0.52 to 0.67 (*p* < 0.001). Stewardship and authenticity exhibit moderate correlations with PS and its subscales, with coefficients ranging from 0.45 to 0.68 (*p* < 0.001). These findings underscore the interrelationship between SL and PS within team dynamics.

### 3.5. Differences Between Main Variables of the Current Study and Sociodemographic Statistics

Differences in SL and PS (Table 3) regarding sociodemographic characteristics were tested using one-way ANOVA, except for gender, which was tested using a *t*-test (*p* > 0.05). The analysis confirmed no significant differences in SL across all socio-demographic characteristics. Thus, SL with “age” (*F* = 1.2, *p*=0.29), SL with “educational level” (*F* = 1.6, *p*=0.29), SL with “marital status” (*F* = 0.9, *p*=0.44), SL with “years of experience” (*F* = 0.7, *p*=0.58), SL with “Working area” (*F* = 1.6, *ρ* = 0.095), and SL with a “hospital type” (*F* = 0.7, *p*=0.93). Finally, SL with “gender” was analyzed using a *t*-test (*ρ* = 0.918).

According to PS, the analysis results indicated that only the “Working area” showed a significant difference with PS (*F* = 2.2, *p*=0.015), while the other socio-demographic characteristics did not exhibit significant differences. Consequently, the results were no significant differences in PS with “age” (*F* = 2.1, *ρ* = 0.82), PS with “educational level” (*F* = 1.3, *ρ* = 0.27), PS with “marital status” (*F* = 2.2, *ρ = *0.09), PS with “years of experience” (*F* = 0.2, *ρ = 0.93*), PS with “hospital type” (*F* = 1.0, *ρ* = 0.38), and lastly PS with “gender” by using *t*-test (*p*=0.793).

### 3.6. PS Predictors

The preliminary test of association (Table 4) showed, as mentioned earlier, that all subscales are significantly correlated; however, “Working area” was the only socio-demographic factor found to be associated with PS (*ρ* = 0.015). Two approaches were used in this section. The first approach involved the model with SL and its subscales, in addition to socio-demographic factors (Model 1), while the second approach included only “Working area” in addition to SL and its subscales (Model 2) to assess the prediction power. The analysis showed that model 1 that contained all socio-demographic factors, SL, and their subscales was significant (*F* 12, 194 = 21.3, ρ ≤  0.001), while model 2 that contains only working area in addition to SL and their subscales was much stronger and significant (*F* 6, 200 = 40.8, *ρ* < 0.001). However, model 1 had a marginally greater *R*-squared value (*R*^2^ = 0.57) than model 2 (*R*^2^ = 0.55), therefore utilizing the parsimonious advantage of the number of factors per model. Model 2 was selected as the final model that explains the predictors of PS because it demonstrates a clear balance between model simplicity and explanatory power.

*R* ^2^ value in model 1 is slightly higher than in model 2; the difference in explained variance was low. Model 2 compares the level of variance using six predictors, as opposed to 12 predictors in model 1. Additionally, model 2 reflects a stronger overall model fit relative to its complexity (*F* = 40.840 vs. 21.297), and fewer variables in model 2 decrease the risk of overfitting and enhance the interpretation of the results. Moreover, model 2 includes the total SL and working area and has an evidence-based practice of the bivariate association. The bivariate correlation between SL and PS was high (*r* = 0.74), and the regression model supported that the isolated SL had a unique contribution when accounting for other variables. The strength of this association suggested that SL acts as a central mediating construct that encompasses the direction effect on PS. Finally, model 2 was considered more prudent and statistically powerful, aligning with the standard regression test principles.

In addition, as mentioned previously in the correlation section, “Working area” was the only socio-demographic factor associated significantly with PS. The magnitude of *R*^2^ = 0.55 indicated that 55% of the variation in PS was explained by model 2. Accordingly, in model 2, regressing “Working area” and SL, along with their subscales, on PS showed that “Working area” as a socio-demographic predictor had no significant effect on PS (*ρ* = 0.305); also, none of the SL subscales had a significant effect on PS. Thus, empowerment (*ρ* = 0.933), humility (*ρ* = 0.587), standing back (*ρ* = 0.831), stewardship (*ρ* = 0.305), and authenticity (*ρ* = 0.606). However, the SL total score was the only significant predictor (*β* = 0.822, *p* < 0.001) of PS. As mentioned above, nurses with a higher perception of SL were more likely to have higher PS scores. Although all other factors were not statistically significant, empowerment, stewardship, and authenticity were identified as risk factors associated with lower PS scores, whereas humility and standing back were found to be protective factors, leading to higher perceptions of PS.

## 4. Discussion

### 4.1. Nurses' Perceptions of the SL Behaviors Among Nurse Leaders at Jordanian Hospitals

This study aimed to explore how nurses perceive SL behaviors in their leaders. The findings indicated that registered nurses had moderate awareness of SL behaviors, with average scores slightly exceeding the midpoint. The moderate perception may reflect the hierarchical leadership style still prevalent in some Jordanian hospitals, where decisions are often made top-down. This might affect nurses who work with patients to recognize SL behavior properly. This finding aligns with another study conducted in Jordan [14] and in Malaysia [48], where healthcare remains largely physician-centered, and leadership behaviors prioritize authority over empowerment. Previous studies have also shown that HCPs and nurses generally have a moderate perception of SL behaviors. These studies provide valuable insights into how SL is perceived in various work environments, highlighting the importance of understanding nurses' perceptions of leadership within healthcare organizations. Numerous studies suggest that the nurses' and HCPs' perceptions of SL behaviors are influenced by factors such as the culture of the healthcare organization, work environment, working hours, duty shifts, self-interest, emotional intelligence, exposure to stressful situations, and leaders' educational level [22, 25, 43, 49]. Moreover, many studies indicated a higher perception of SL behaviors among their leaders than the current study's results [21, 24, 27, 44]. A study conducted in India found that a higher nurse perception of SL behavior is crucial in the healthcare sector to maintain a high-quality level of care [26].

### 4.2. The Nurses' Perceptions of Their PS Levels at Jordanian Hospitals

This study addresses the second research question regarding nurses' perceptions of PS. The results indicate that the nurses had a moderate perception of PS and were close to the upper limit of the scale. Despite the PS score being close to the upper limit, the moderate PS level may reflect the lack of open communication and discussion, fear of punishment for incident reporting, or limited participation of nurses in decision-making. The finding may also reflect that the leaders' behaviors support their team, given the moderate score; however, this score does not translate to full PS. These results are in line with previous studies that indicated that nurses and HCPs in hospitals are likely to perceive PS moderately; overall, all these studies confirmed that nurses' and HCPs' perceptions of PS are an essential aspect in healthcare institutions [21, 38, 40, 41]. In contrast to the current study result, two studies found that the PS perception among HCPs was high [15, 50]. Variations in nurses' perceptions of PS may be attributed to several factors, including the participants' country and organizational cultures, leaders' behaviors, organizational rules and regulations, and the presence of a stressful work environment. By analyzing the results of this study, it is now possible to gain a deeper understanding of how nurses view PS.

### 4.3. Relationship Between SL and PS

Based on the current study's findings, a substantial and affirmative relationship was observed between the practice of SL and the PS of nurses in the workplace. This result indicates that leaders who prioritize serving their team and empowering them tend to create a work environment where nurses feel safe expressing themselves and sharing their ideas and concerns without fear of retribution or criticism. Previous studies have reported a strong positive correlation between SL and PS, concluding that SL promotes PS among nurses and healthcare professionals to foster a healthcare environment [21, 43]. Another study found a moderately positive relationship between SL and PS [42, 51]. This relationship aligns with the social exchange theory, which posits that supportive leadership fosters trust, promotes mutual respect, and reduces interpersonal risk [52].

In a study by Sasaki et al., the analysis of the SL subscale revealed that empowerment had the strongest positive correlation with PS, followed by stewardship, authenticity, and standing back. However, humility showed a moderate and significant positive correlation. Empowerment in this study was consistent with that mentioned in a study conducted in Japan [41], but the other subscales differed. Humility was the second strongest subscale, followed by standing back, authenticity, and lastly, stewardship. Empowerment showed the strongest correlation, possibly because it directly impacts nurses' autonomy and decision-making confidence. In contrast, stewardship may be perceived as the less frequent use of it in daily interactions.

A previous study examining the correlation between the SL total scale and PS subscales found a strong, positive correlation between the PS subscale and the team leader. However, weak correlations were observed for the subscales “in relation to peers” and in relation to the team as a whole [44]. Another study examined the correlation between the SL subscale and PS subscale and found that the empowerment subscale showed a strong, positive, and significant correlation with “in relation to team leader” and “in relation to team as a whole” PS subscales and had a moderate, positive, and significant correlation with “in relation to peers” PS subscale. Furthermore, this previous study found a strong, positive, and significant correlation between SL subscales and PS subscales “in relation to team leaders” but had a moderate, positive, and significant correlation among humility, stewardship, standing back, and authenticity, “in relation to the team as a whole” and “in relation to peers” [41].

The current study revealed that all SL subscales exhibited strong, positive, and significant correlations with the corresponding PS subscales, except for the relationship between the stewardship and authenticity SL subscale and the “in relation to peers” PS subscale, which showed a moderate, positive, and significant correlation. The variation in country and organizational culture explains the consistency or contradiction of the current study with previous studies.

### 4.4. Predictors of PS

The current study's results indicated that SL explained PS and was a significant predictor of PS. These findings suggest that leaders in healthcare organizations should strive to create a psychologically safe environment for their nurses' followers by adopting a servant leadership (SL) approach. This study confidently predicts a moderate direct effect of SL on PS, aligning well with previous research findings [21, 42, 43]. SL behaviors, such as standing back and empowering nurses, may minimize the fear of punitive consequences, especially in high-power hospital environments, thereby improving PS.

### 4.5. Clinical Implications

Effective leadership creates a safe environment that allows followers to express their ideas, concerns about their work, and issues related to patients or incidents that have occurred without fear of retribution or criticism. By promoting a culture of PS, leaders might encourage effective teamwork and collaboration, leading to improved quality of care outcomes by learning from their mistakes. Furthermore, leaders might foster learning behaviors by providing opportunities for feedback, continuous improvement, and skills development [15, 37]. The findings suggest that hospital administrators should integrate the SL style into management training programs. Focusing on listening ability, empathy, and empowerment are considered vital to foster PS.

### 4.6. Limitations

Longitudinal research design is an effective approach that could significantly improve the accuracy of results obtained and may help researchers identify changes in variables over time, determine the causal relationship between variables, and identify trends or patterns that may not be apparent in cross-sectional studies. In the context of hospitals, it is essential to conduct additional studies that focus on the causality of differences among hospital departments. The variations in leadership styles, management practices, and infrastructure might significantly impact the overall performance of the departments. By identifying the factors that contribute to these differences, hospital administrators could implement changes that improve the quality of care provided to patients.

Convenience sampling techniques can be complex and may impact the generalizability of the results, but they offer an easy-to-implement and cost-effective alternative. This method does not require a list of all population elements, making it quick and accessible [53].

## 5. Conclusion

This study highlighted that nurses in Jordanian hospitals perceiving SL behaviors among their leaders at a moderate level while also reporting a moderate yet near the upper limit level of PS. The strong positive correlation between SL and PS highlights the crucial role of leadership in creating a supportive work environment where nurses feel secure in expressing themselves without fear of negative consequences. However, variation between studies suggests that organizational culture, work environment, and leadership practices significantly affect these perceptions. Ultimately, to enhance nurse well-being and patient care outcomes, healthcare institutions should prioritize leadership development programs that cultivate SL qualities, thereby strengthening PS within nurse HCPs. In the future, further studies are needed to examine the contextual and cultural factors that shape these dynamic and diverse healthcare settings.

## Figures and Tables

**Table 1:** Servant leadership and psychological safety survey descriptive statistics.

Variable	M	SD	MD	Min	Max	P_25_	P_75_
*Servant leadership survey descriptive statistics (N = 207)*							
Servant leadership total score	66.7	18.4	69	18	108	51	81
Empowerment	22.8	6.9	23	6	36	18	29
Humility	10.8	3.5	11	3	18	8	13
Standing back	11	3.2	12	3	18	9	13
Stewardship	11.2	3.6	12	3	18	9	14
Authenticity	10.8	3.7	11	3	18	8	14

*Psychological safety survey descriptive statistics (N = 207)*							
Total psychological safety	90.4	21.8	93	19	133	77	108
“In relation to the nurses' team leader”	42.7	11.5	45	9	63	36	52
“In relation to nurses' peers/the other members of their team”	33	8.6	35	7	49	27	40
“In relation to the team as a whole”	14.7	3.9	16	3	21	12	18

**Table 2:** Correlations between servant leadership and psychological safety and their subscales.

Variables	Total servant leadership	Empowerment	Humility	Standing back	Stewardship	Authenticity
Total psychological safety	0.74	0.68	0.65	0.64	0.62	0.63
Psychological safety in relation to the team leader	0.77	0.71	0.67	0.64	0.64	0.68
Psychological safety in relation to peers	0.57	0.54	0.52	0.52	0.47	0.45
Psychological safety in relation to the team as a whole	0.61	0.55	0.52	0.54	0.57	0.50

*Note:* Correlation is significant at alpha < 0.05 (2-tailed).

**Table 3:** Differences in servant leadership and psychological safety regarding demographic characteristics.

**Differences in servant leadership and psychological safety regarding demographic characteristics**						
**Variable**	**Servant leadership**			**Psychological safety**		
	***F***	**p** ^∗^		***F***	**p** ^∗^	

Age	1.2	0.293		2.1	0.820	
Educational level	1.6	0.204		1.3	0.274	
Marital status	0.9	0.436		2.2	0.085	
Years of experience	0.7	0.583		0.2	0.930	
Working area	1.6	0.095		2.2	0.015	
Hospital type	0.7	0.932		1.0	0.384	

**Differences in servant leadership and psychological safety in relation to**gender^**a**^						
**Variables**		***N***	**M**	**SD**	***t*-test**	**p** ^∗^

Servant leadership	Male	75	89.9	20.6	−0.263	0.793
	Female	132	90.7	22.6		

Psychological safety	Male	75	66.5	18.9	−0.103	0.918
	Female	132	66.8	18.2		

*Note:* A one-way repeated measures' ANOVA was applied. The multivariate normality assumption is fulfilled.

^∗^Significance at 0.05.

^a^Independent sample *T*-test.

**Table 4:** Predictors of psychological safety using standardized regression.

**Psychological safety**predictors^∗^						
***R***	**R** ^2^	**Adjusted** **R** ^2^	**Std. error of the estimate**	***F***	**df1**	**df2**

Model 1						
0.754^a^	0.568	0.542	14.78956	21.297	12	194
Model 2						
0.742^b^	0.551	0.537	14.86446	40.840	6	200

**Regressing “working area” and SL, and their subscales on total PS (N = 207)**						
**Variable**		***B***	**SE**	***β***	**p**	

Working area		−0.322	0.313	−0.050	0.305	
Empowerment		−0.012	0.139	0.004	0.933	
Humility		0.333	0.611	0.053	0.587	
Standing back		0.138	0.646	0.020	0.831	
Stewardship		−0.653	0.635	−0.108	0.305	
Authenticity		−0.303	0.587	−0.051	0.606	

*Note:* β: standardized beta, B: unstandardized beta, *ρ*: probability, and SE: standard error for the unstandardized beta. Normality assumptions of normality, independence, homoscedasticity, and linearity are fulfilled.

^∗^Standardized regression.

^a^Predictors: (constant), total servant leadership, age, educational level, working area, hospital type, gender, marital status, experience in years, total mean of humility, total mean of standing back, total mean of authenticity, and total mean of stewardship.

^b^Predictors: (constant), total servant leadership, working area, total mean of humility, total mean of standing back, total mean of authenticity, and total mean of stewardship.

## Data Availability

The data that support the findings of this study are available on request from the corresponding author. The data are not publicly available due to privacy or ethical restrictions.
